# The Neuronal Functions of EF-Hand Ca2+-Binding Proteins

**DOI:** 10.3389/fnmol.2012.00092

**Published:** 2012-09-11

**Authors:** Michael R. Kreutz, Jose R. Naranjo, Karl-Wilhelm Koch, Beat Schwaller

**Affiliations:** ^1^Leibniz-Institute for NeurobiologyMagdeburg, Germany; ^2^Centro Nacional de Biotecnología, Consejo Superior de Investigaciones CientíficasMadrid, Spain; ^3^Institute of Biology and Environmental Science, Carl von Ossietzky University OldenburgOldenburg, Germany; ^4^Department of Medicine, University of FribourgFribourg, Switzerland

Neuronal Ca^2+^ signaling exhibits highly restricted and dynamic gradients called Ca^2+^ waves, spikes, transients, and puffs depending upon their corresponding spatial and temporal features. The central role of Ca^2+^ in cellular physiology of neurons is based on a Ca^2+^-signaling toolkit that assembles intracellular signaling systems with different spatial and temporal dynamics (Berridge, 2000). The cytosolic Ca^2+^ concentration is tightly regulated by binding and chelation of the ion by various Ca^2+^-binding proteins (CaBPs) and by transport of the ion across plasma and intracellular membranes. The complex regulation of cytosolic Ca^2+^ concentrations is the subject of an increasing number of investigations, because this regulation is intimately linked to the function of Ca^2+^ in neurotransmitter release, synaptic plasticity, neurosensory signaling, activity-dependent gene transcription, intracellular trafficking, and many other cellular processes. The multitude of Ca^2+^-regulated processes requires specialized downstream processing machinery, translating the Ca^2+^ signal into alterations of cellular functions. It is generally believed that the versatile basis for the complex signaling in micro- and nanodomains of neuronal subcompartments is provided by the existence of a multitude of different CaBPs from which numerous belong to the EF-hand super-family. EF-hand proteins are traditionally subdivided into Ca^2+^ buffer and Ca^2+^ sensor proteins. This distinction is however not really valid, because Ca^2+^-binding to EF-hand proteins can serve both functions, even at the same time. Nonetheless, whereas the first group is characterized by a rather high affinity for Ca^2+^, exhibits little conformational change upon Ca^2+^-binding and is thought to mainly chelate Ca^2+^, the second group has a somewhat lower affinity for Ca^2+^ (often in the 1–10 μM range) and shows considerable conformational changes upon Ca^2+^-binding, which usually triggers a target interaction.

Members of the latter group belong either to the Neuronal calcium sensor (NCS) proteins or the related Caldendrin/CaBP/Calneuron (nCaBPs) family. All of these proteins resemble to a varying degree to the structure of their common ancestor Calmodulin (CaM), but they are quite diverse in amino acid sequence in comparison to CaM. It is therefore surprising that relatively few binding partners for NCS/nCaBP proteins have been identified that are not also CaM targets and this raises the question on the specificity and function of these interactions. Interestingly, binding of target proteins to NCS proteins and nCaBP has frequently different consequences than binding to CaM, which substantially increases the versatility of the Ca^2+^ signaling toolkit. The general idea of this special issue was to provide an overview on the function of neuronal EF-hand CaBPs in health and disease. The issue contains reviews that summarize the state-of-the-art in the field, as well as experimental and theoretical papers dealing with emerging concepts on Ca^2+^-signaling/buffering mediated by EF-hand CaBPs. Questions like which features define the functional role of a EF-hand Ca^2+^ sensor in neurons, the conditions under which a given interaction of a CaBP with its target is of physiological relevance, the emerging synaptic role of these proteins, and mounting evidence for their role in the regulation of protein trafficking are covered. Structural aspects and biophysical studies are included and provocative new ideas based on numerical modeling are part of this issue. Another interesting aspect covered in the research topic is the emerging role of CaBPs in brain disease states. Several papers have shown that CaBPs are targets of drugs in clinical use, that expression levels of CaBPs are frequently altered in brain disease states, and more recently reports on mutations in EF-hand Ca^2+^ sensors linked to human disease were published. We want to thank all authors for the high quality of the papers that they have submitted and the efforts that they have made to provide an excellent overview of this interesting field. It was a pleasure to edit this research topic.
